# Three-Dimensional S/CeO_2_/RGO Composites as Cathode Materials for Lithium–Sulfur Batteries

**DOI:** 10.3390/ma11091720

**Published:** 2018-09-14

**Authors:** Qiuyan Hao, Guoliang Cui, Yuan Tian, Taizhe Tan, Yongguang Zhang

**Affiliations:** 1 School of Materials Science and Engineering, Research Institute for Energy Equipment Materials, Hebei University of Technology, Tianjin 300130, China; haoqiuyan@hebut.edu.cn (Q.H.); cgl19951120@163.com (G.C.); tianyuanhebut@163.com (Y.T.); 2 Synergy Innovation Institute of GDUT, Heyuan 517000, China; taizhetan@gdut.edu.cn

**Keywords:** Lithium–sulfur battery, CeO_2_/RGO composite, electrochemical performance

## Abstract

In this paper, the synthesis of the three-dimensional (3D) composite of spherical reduced graphene oxide (RGO) with uniformly distributed CeO_2_ particles is reported. This synthesis is done via a facile and large-scalable spray-drying process, and the CeO_2_/RGO materials are hydrothermally compounded with sulfur. The morphology, composition, structure, and electrochemical properties of the 3D S/CeO_2_/RGO composite are studied using X-ray diffraction (XRD), scanning electron microscope (SEM), transmission electron microscopy (TEM), thermal gravimetric analysis (TGA), Raman spectra and X-ray photoelectron spectroscopy (XPS), etc. The electrochemical performance of the composites as electrodes for lithium–sulfur batteries is evaluated. The S/CeO_2_/RGO composites deliver a high initial capacity of 1054 mAh g^−1^, and retain a reversible capacity of 792 mAh g^−1^ after 200 cycles at 0.1 C. Profiting from the combined effect of CeO_2_ and RGO, the CeO_2_/RGO materials effectively inhibit the dissolution of polysulfides, and the coating of spherical RGO improves the structural stability as well as conductivity.

## 1. Introduction

With the booming use of electric vehicles and portable electronic devices, the demand for rechargeable batteries that have higher power densities and long-term stability has increased substantially [1,2]. Lithium–sulfur batteries are secondary batteries that have high-energy current density (2600 Wh kg^−1^), as well as great potential for development and application prospects [3]. In addition, in terms of source, cost, and environmental impact, sulfur has also been shown to have unparalleled advantages for being used as a positive electrode [4,5]. However, lithium–sulfur batteries still have some shortcomings [6,7]. First, sulfur insulation reduces the use of cathode-active materials. Second, a large volume change (80%) is produced during charging/discharging, which leads to reduced mechanical properties. Third, the dissolution of polysulfides leads to a shuttle effect between the cathode and anode, and this results in the loss of active materials and poor coulomb efficiency, poor utilization, and obvious degradation [8].

Numerous design methods, including the combination of sulfur and carbon materials [9,10,11], metal oxides [12,13], and conductive polymers [14,15], have been explored to avoid these problems. Among these materials, reduced graphene oxide (RGO) (which is a carbon material) has high surface area, excellent intrinsic conductivity, excellent mechanical flexibility, and chemical stability. Due to these excellent properties, RGO has been widely used to prepare S/RGO composites to mitigate the dissolution of intermediate polysulfides [16,17]. However, the physical interactions between nonpolar RGO and polar polysulfides are weak, and they cannot ensure the long-term confinement of polysulfides during the charging/discharging process, during which the polysulfides remain vulnerable to slow dissolution in electrolytes, thus triggering the “shuttle effect” and resulting in an unsatisfactory calendar life [18].

Polar materials can be firmly combined with polysulfides via chemical adsorption, and thus polysulfides can be effectively captured at the cathode. Many polar host materials for sulfur, including SiO_2_, TiO_2_, Al_2_O_3_, La_2_O_3_ and MnO_2_, have thus far been introduced into the cathodes [19,20,21]. For example, Sun et al. reported a method of modifying nitrogen-rich mesoporous carbon using La_2_O_3_ nanodots [22]. Their results show that the La_2_O_3_ nanoparticles can be used as the adsorption point of polysulfides and oxidation-reduction catalyst. Ding et al. fabricated nanoscale graphene modified with TiO_2_ nanocrystals and used it as the sulfur host [23]. The TiO_2_ nanocrystals can adsorb dissolved polysulfides and also promote the transmission of charge. CeO_2_, which is a polar substance, is also an excellent adsorbent and catalyst. CeO_2_ has been applied to the preparation of cathode materials for lithium sulfur batteries. In addition to effectively slowing down the dissolution of polysulfides in electrolytes, CeO_2_ also has a catalytic effect on the redox reaction. However, the conductivity of CeO_2_ is relatively low, which inevitably affects the electrochemical performance.

Herein, a simple and large-scale spray-drying technique has been used to prepare RGO coated with CeO_2_ particles. The CeO_2_/RGO composites have several apparent advantages. First, spherical RGO greatly improves the conductivity of the electron and ion transmission during the charging/discharging process. In addition, CeO_2_ particles provide several strong binding sites for polysulfide intermediates, and keep them bound to the cathode materials during the charging/discharging process, which results in a longer cycle life. Therefore, the S/CeO_2_/RGO cathodes have the advantages of a high reversible capacity, good multiplying performance, and good circulation stability.

## 2. Materials and Methods

### 2.1. Materials

All of the chemicals that were used were analytical grade and used without further purification. Cerium nitrate hexahydrate (Ce(NO_3_)_3_·6H_2_O), ammonia solution ((NH_3_·H_2_O), graphene oxide solution (GO), polyvinylidene fluoride (PVDF), and N-methyl-pyrrolidinone (NMP) were purchased from Shanghai Aladdin Bio-Chem Technology Co., Ltd. (Shanghai, China).

### 2.2. Sample Preparation

**Figure 1:**
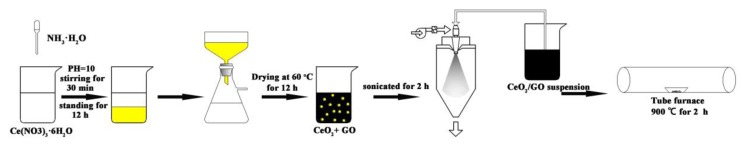
Schematic diagram of the fabrication process of CeO_2_/reduced graphene oxide (RGO) composites.

CeO_2_ was synthesized via a precipitation process. Ammonia solution (NH_3_·H_2_O) was added dropwise to an aqueous solution of Ce(NO_3_)_3_·6H_2_O solution, which had a concentration 0.4 mol L^−1^, until the pH of the mixture became 10. After stirring for 30 min, the mixture was then left standing for 12 h. The precipitate was filtered out of the solution using a filtration device, and then it was repeatedly washed with water. Afterward, the samples were desiccated at 60 °C for 12 h in an electronic oven. The sample was then calcined at 300 °C for 4 h in a muffle furnace to obtain the desired CeO_2_. The second step was to composite CeO_2_ and RGO. A commercially available graphene oxide (GO) solution (2 mg mL^−1^) was mixed with CeO_2_ in ratio of 1:5. The mixture was sonicated for 2 h at 50 kHz using an ultrasonic cell crusher at room temperature to obtain a uniformly mixed suspension of CeO_2_/GO. The spray-drying technique was then used to obtain CeO_2_/GO powders. The spray-drying equipment that was used was a normal air pressurizer with an inlet air temperature of 200 °C and a feed rate of 4 mL min^−1^. The precursor was calcined in a tube furnace under an argon atmosphere at 900 °C for 2 h to achieve the CeO_2_/RGO composites (Figure 1). In the final step, sulfur was loaded into the CeO_2_/RGO composite. The weight ratio of CeO_2_/RGO to sulfur was set to 1:2. The mixture was heated at 155 °C, and maintained at this temperature for 12 h to obtain the S/CeO_2_/RGO composites. The chemical equations associated with the preparation of CeO_2_ are as follows:

(a) Ce^3+^ + 3OH^−^→Ce(OH)_3_↓

(b) Ce(OH)_3_ + 1/4O_2_ + 1/2H_2_O→Ce(OH)_4_

(c) Ce(OH)_4_→CeO_2_ + 2H_2_O

### 2.3. Characterization

Morphology and crystal structure information were acquired using scanning electron microscopy (SEM, Rigaku S4800, Neu-Isenburg, Germany), transmission electron microscopy (TEM, TECNAI F-20, Thermo Fisher Scientific, Waltham, MA, USA), and X-ray diffraction (XRD, D/max-rB, Rigaku, Toyko, Japan). The surface functional groups in the S/CeO_2_/RGO composites were determined using a Physical Electronics PHI 5700 spectrometer (Chanhassen, MN, USA). The pyrolysis weight analysis (TGA) was performed using a Mettler Toledo-TGA/DSC (HK).

### 2.4. Electrochemical Measurements

S/CeO_2_/RGO, acetylene black, and PVDF were mixed using magnetic stirring in a weight ratio of 8:1:1 with *N*-methylpyrrolidone (NMP) as a solvent to prepare the cathode slurry. N-methylpyrrolidone (NMP) was slowly added to the materials and ground until a similar viscous oil-like slurry was formed. The obtained slurry was then cast on aluminum foil and dried at 60 °C for 12 h in vacuum, and the NMP evaporated completely during the drying process. Aluminum foil was cut into disks, each with a diameter of 15 mm, for use as current collectors. The electrolyte was 1 M of lithium bis (trifluoromethane)sulfonimide (LiTFSI) in a mixed solvent of 1,2-dimethoxyethane (DME) and 1,3-dioxolane (DOL) (1:1 *v*/*v*) containing 1 wt % of LiNO_3_. Cyclic voltammetry (CV) and electrochemical impedance spectroscopy (EIS) were conducted using an electrochemical workstation (CHI660E, Austin, TX, USA) that was operated in the frequency range of 10 kHz to 10 mHz with an amplitude of 10 mV.

## 3. Results and Discussion

**Figure 2:**
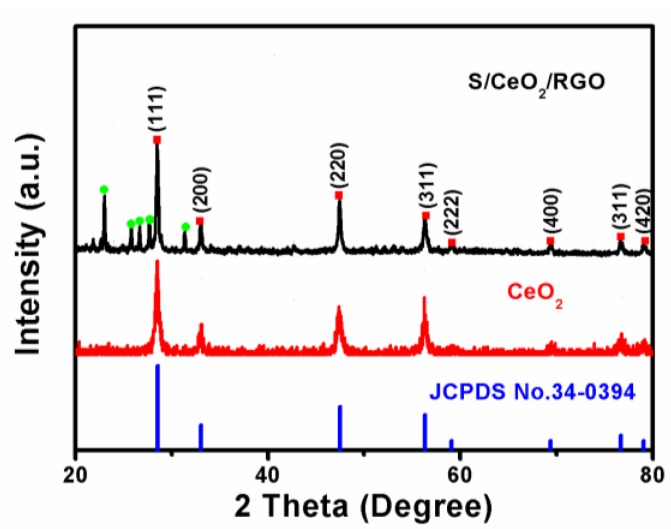
XRD patterns of CeO_2_ and S/CeO_2_/RGO.

Figure 2 shows the XRD patterns of CeO_2_ and S/CeO_2_/RGO composites. XRD peaks were recorded at 2*θ* = 28.5°, 33.1°, 47.4°, 56.3°, 69.4°, 76.6° and 79.0°, and could be well allocated to the (111), (200), (220), (311), (400), (331) and (420) planes, respectively, of CeO_2_ (JCPDS No. 34-0394) [24]. Two feeble peaks of 3D RGO are observed at 26.2° and 43.7° because of a fairly low diffraction intensity of 3D RGO [25]. The other peaks are sulfur peaks (JCPDS No. 42-1278) [26]. A few strong peaks are marked in the figure.

**Figure 3:**
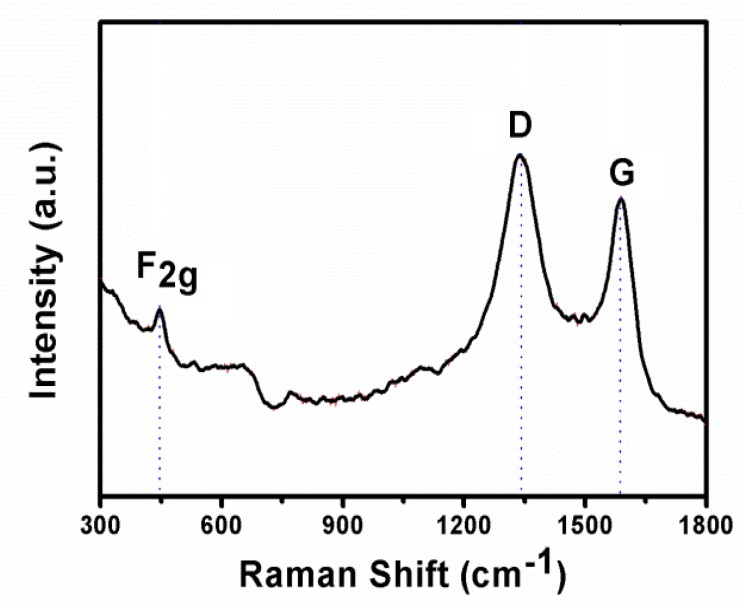
Raman spectra obtained from CeO_2_/RGO composites.

To further confirm the structural intricacies present in the CeO_2_/RGO composites, we collected Raman spectra, and the results are shown in Figure 3. All of the CeO_2_/RGO composites exhibited an inherent mode of graphite structure (D-breathing zone at ~1350 cm^−1^ and G-breathing zone at ~1580 cm^−1^) and CeO_2_ structure (F_2g_ mode at ~461 cm^−1^) [27]. The degree of graphitization in the CeO_2_/RGO composites is low, because the addition of metal oxide leads to an increase in the ratio I_D_:I_G_, thereby increasing the defect level of graphene and increasing the conductivity of graphene [28].

**Figure 4:**
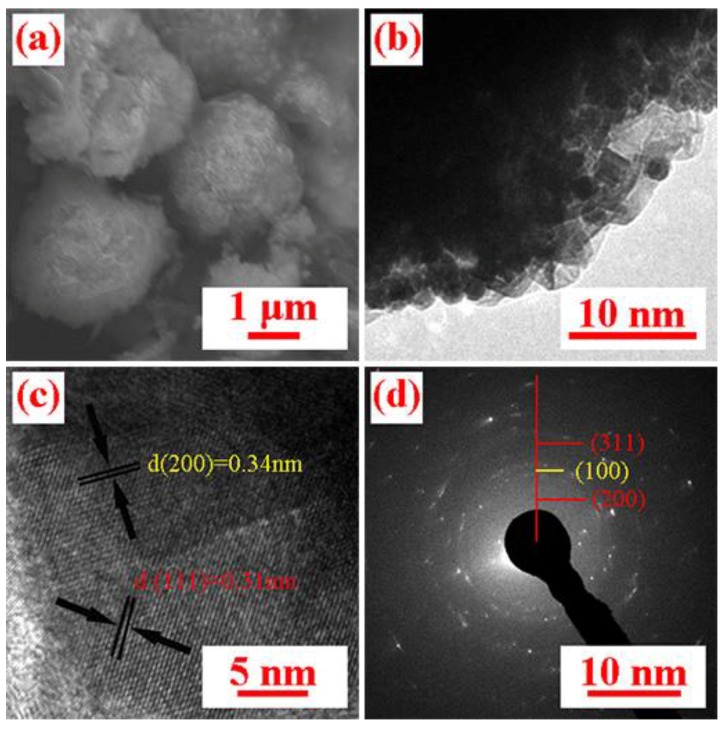
(**a**) SEM image of CeO_2_/RGO. (**b**) TEM image of CeO_2_/RGO. (**c**) High-resolution transmission electron microscopy (HRTEM) image of CeO_2_/RGO. (**d**) SAED pattern of CeO_2_/RGO.

As seen in the SEM and TEM images of the CeO_2_/RGO sample (Figure 4a,b), RGO has been made into a three-dimensional (3D) spherical structure via spray-drying, and CeO_2_ was distributed uniformly in the RGO. In the corresponding high-resolution TEM image shown in Figure 4c, RGO has lattice spacings of ca. 0.34 nm, which is indexed to the (200) planes, and ca. 0.312 nm, which corresponds to the interspacing of the (111) planes of cubic CeO_2_ [27]. The selective electron diffraction (SAED) pattern of the composites materials reveals the polycrystalline nature of the materials (Figure 4d) [29]. The above results show that CeO_2_ and RGO are well combined to form composite materials.

**Figure 5:**
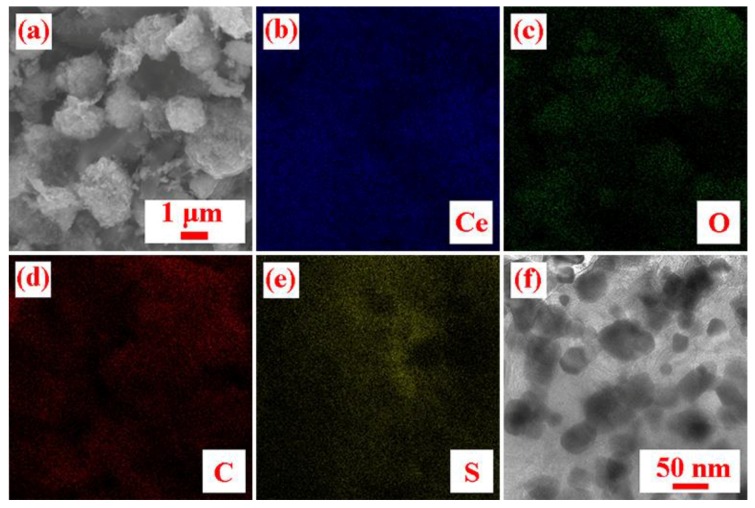
(**a**) SEM image of S/CeO_2_/RGO. (**b**–**e**) Element mapping of S/CeO_2_/RGO. (**f**) TEM image of S/CeO_2_/RGO.

SEM and TEM images of the S/CeO_2_/RGO sample are shown in Figure 5a,f, respectively. As seen in the figure, the resulting sphere has a diameter of about 1–2 µm. Additionally, the element mapping results (Figure 5b–e) reveal that Ce, O, C, and S are distributed throughout the structure, indicating the component uniformity of the S/CeO_2_/RGO composites.

**Figure 6:**
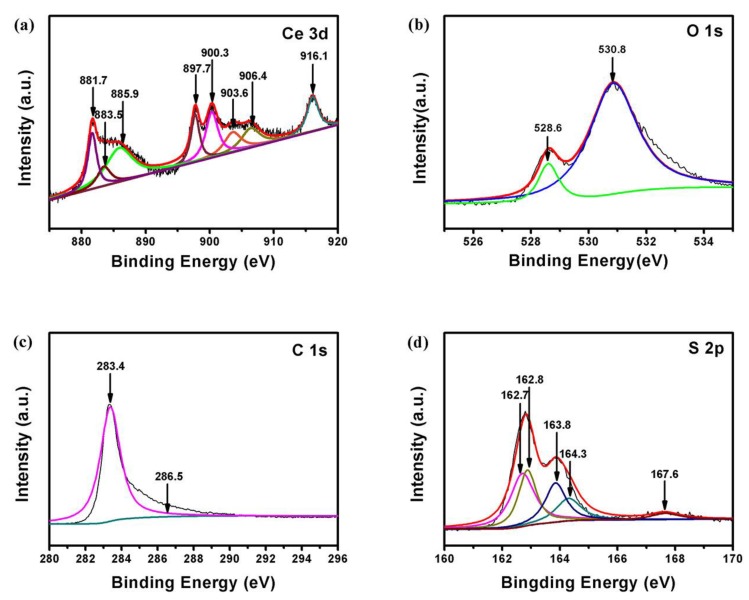
X-ray photoelectron spectroscopy (XPS) binding energy spectra of the core level of (**a**) Ce, (**b**) O, (**c**) C and (**d**) S in the resulting samples.

The high-resolution XPS spectrum of 3D Ce is shown in Figure 6a, and demonstrates the presence of a mixed valence state. The O 1s XPS peak at 530.8 eV corresponds to the oxygen in CeO_2_, and further confirms the presence of CeO_2_ (Figure 6b) [24]. The O 1s peak at 528.6 eV indicates that there are residual oxygen groups associated with the C atoms in 3D RGO. The C 1s XPS spectrum of S/CeO_2_/RGO is shown in Figure 6c. The peak observed at 283.34 eV is related to the graphitic carbon in the 3D RGO, and the peak at 286.48 eV is assigned to the C–O bond [30]. In Figure 6d, the binding energies of S 2p_3/2_ are 163.8 and 164.3 eV, and are attributed to the S–S and S–O species, respectively [30]. The additional small shoulder of 167.7 eV is attributed to the sulfate species, which is associated with sulfur oxidation [31].

**Figure 7:**
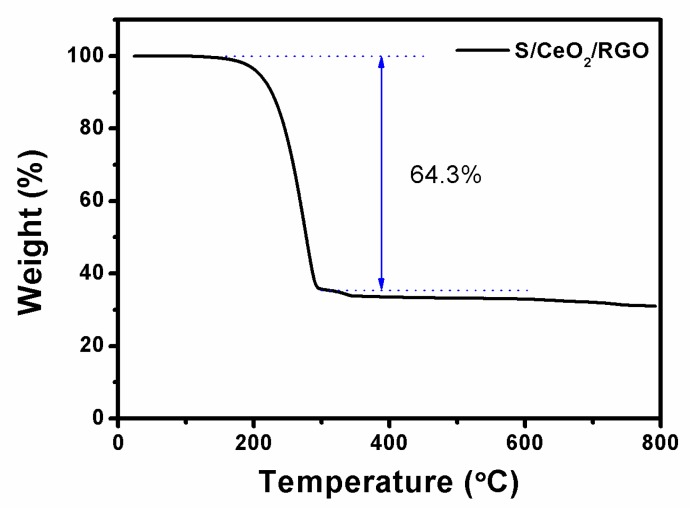
TGA curve of S/CeO_2_/RGO at a heating rate of 10 °C /min.

It is apparent from the TGA curves shown in Figure 7 that the weight drops rapidly when the temperature increases from 200 °C to 293 °C. Since the sulfur is completely evaporated [32], the rapid weight loss is about 64 wt %. Therefore, the overall sulfur content can be estimated to be about 64 wt %.

**Figure 8:**
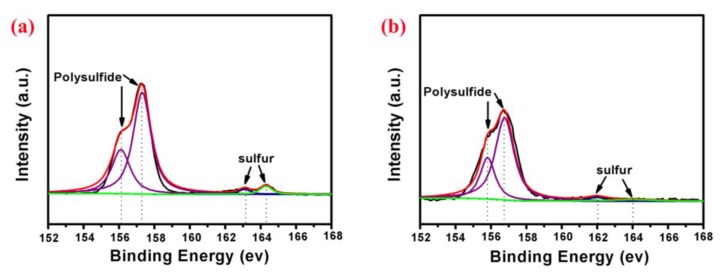
S 2p XPS spectra of the (**a**) S/CeO_2_/RGO and (**b**) S/RGO cathodes after 200 cycles.

The S/CeO_2_/RGO and S/RGO cathodes were tested after 200 cycles, and the S 2p XPS spectra of the two samples are shown in Figure 8. There are four apparent peaks for each sample. For S/CeO_2_/RGO, these are at 156.2 eV, 157.3 eV, 163.1 eV and 164.3 eV. For S/RGO, they are at 155.7 eV, 156.8 eV, 162.1 eV, and 164 eV. For both samples, the peaks around 156 eV correspond to lithium polysufides, and the peaks around 163 eV correspond to elemental sulfur. The S 2p XPS spectra of the S/CeO_2_/RGO cathode after cycling obviously show higher binding energies compared with those of the S/RGO cathode. Therefore, the CeO_2_ particles embedded in spherical RGO can serve as strong adsorbents of lithium polysulfides, which in turn improve the electrochemical characteristics.

**Figure 9:**
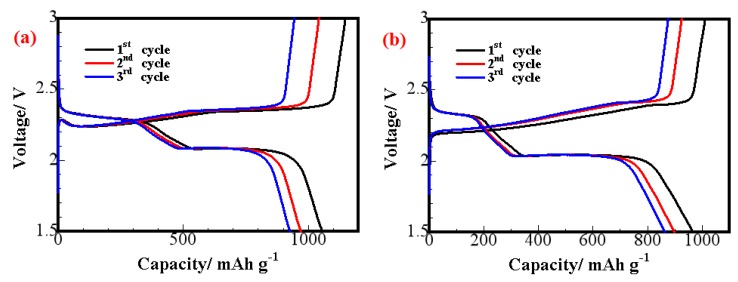
Charging/discharging curves of the lithium–sulfur batteries with the (**a**) S/CeO_2_/RGO and (**b**) S/RGO cathodes at 0.1 C.

Figure 9 shows the charge/discharge curves for lithium–sulfur with the S/CeO_2_/RGO and S/RGO cathodes at a scan rate of 0.1 C. In the discharge process with the S/CeO_2_/RGO cathode, two major stages appear in the potential distribution, which are attributed to the two-step electrochemical reaction between lithium and sulfur. A short discharge platform of about 2.3 V indicates the first electrochemical reaction, and is related to the reduction of the S_8_ form of elemental sulfur [33]. The lower extended plateau around 2.1 V in the discharge curve reflects the subsequent reduction of higher polysulfides to lower polysulfides, and eventually to lithium sulfide Li_2_S [32]. The S/CeO_2_/RGO electrode presents a higher initial discharge capacity than the S/RGO electrode during discharge at 0.1 C. Meanwhile, the S/CeO_2_/RGO electrode shows two higher discharge potential plateaus than the S/RGO electrode. These are all because CeO_2_ decoration enhances catalytic activity.

**Figure 10:**
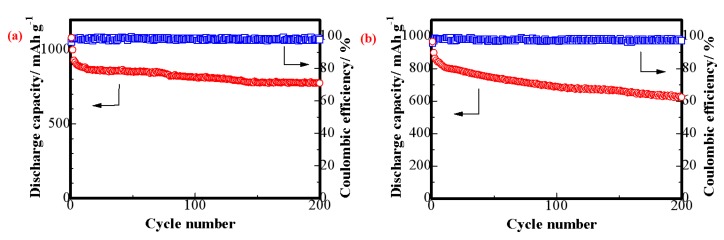
Cycling performances (red) and coulombic efficiencies (blue) of the lithium–sulfur batteries with the (**a**) S/CeO_2_/RGO and (**b**) S/RGO cathodes under 0.1 C.

As seen in Figure 10a, the cycle performances of batteries with the S/CeO_2_/RGO cathode were measured under 0.1 C. The initial discharge capacity was 1054 mAh g^−1^, corresponding to a sulfur utilization of 65%. Furthermore, the S/CeO_2_/RGO cathodes enhanced the cyclability of the batteries, retaining a discharge capacity of 792 mAh g^−1^ even after 200 cycles. On the contrary, the S/RGO cathode (Figure 10b) delivered a lower discharge capacity of approximately 965 mAh g^−1^ at the same current rate. After 200 cycles, the discharge capacity quickly decreased to 623 mAh g^−1^. The coulombic efficiency of the batteries with the S/CeO_2_/RGO cathode was close to 100%, whereas the coulombic efficiency of the S/RGO cathode was lower than 98%, indicating that the soluble polysulfides from the cathodes were largely adsorbed by the S/CeO_2_/RGO materials.

**Figure 11:**
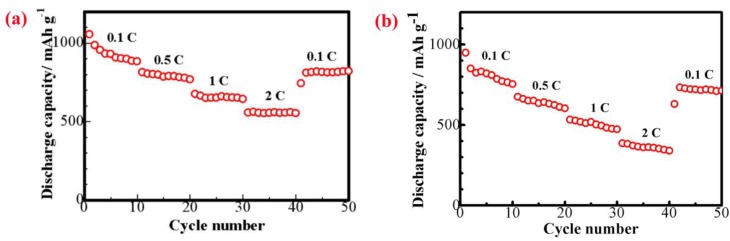
Rate performances of lithium–sulfur batteries with the (**a**) S/CeO_2_/RGO and (**b**) S/RGO cathodes at different current densities.

Figure 11 shows the rate capability at different current densities of the S/CeO_2_/RGO and S/RGO cathodes. As the current density increased from 0.1 C to 2 C, the discharge capacity changed steadily; under 0.1 C, 0.5 C, 1 C, and 2 C, for S/CeO_2_/RGO, the reversible capacities were 1054 mAh g^−1^, 807 mAh g^−1^, 674 mAh g^−1^, and 552 mAh g^−1^, respectively, and for S/RGO, the reversible capacities were 948 mAh g^−1^, 680 mAh g^−1^, 512 mAh g^−1^ and 394 mAh g^−1^, respectively. Apparently, the discharge capacities of the S/CeO_2_/RGO cathode at each current rate were larger than those of the S/RGO cathode. Moreover, when the current rate returned to 0.1 C, S/CeO_2_/RGO remains almost at capacity. This is ascribed to the absorbing and catalyzing effects of CeO_2_ particles on lithium polysulfides during the redox procedures [26].

**Figure 12:**
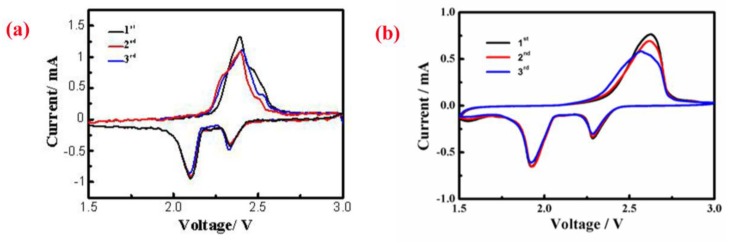
Cyclic voltammetry (CV) curves of the (**a**) S/CeO_2_/RGO and (**b**) S/RGO cathodes at a scan rate of 0.1 mV s^−1^.

As presented in the Figure 12, both the S/CeO_2_/RGO and S/RGO cathodes display two obvious cathodic peaks and one anodic peak during the cathodic sweep; the peaks at 2.3 and 2.1 V are attributed to the change of elemental sulfur into soluble lithium polysulfide. In the subsequent anodic scan, the obvious peak at 2.4 V corresponds to Li_2_S_8_ [34]. Compared with the S/RGO sample, the S/CeO_2_/RGO sample has a higher charge/discharge peak, which verifies the rapid electron/ion transfer and redox process [35]. The cathode peak potential of the S/CeO_2_/RGO cathode is about 2.1 V; this is slightly larger than the cathode peak potential of the S/RGO cathode, which is about 1.9 V. The relatively larger cathodic peak potential indicates that the sulfur in the cathode electrode can react with Li ions more easily because of the decoration of CeO_2_ particles, which demonstrates the catalytic effect of CeO_2_.

**Figure 13:**
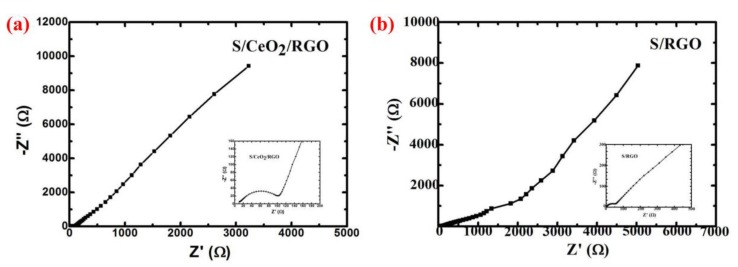
Electrochemical impedance spectroscopy (EIS) Nyquist plots of the (**a**) S/CeO_2_/RGO and (**b**) S/RGO electrodes.

To gain further insight into the reaction kinetics, the charge transfer resistance (Rct) of the S/CeO_2_/RGO and S/RGO cathodes was examined with EIS data (Figure 13). The EIS data demonstrate a semicircle in the medium frequency region and a tail with a slope in the lower frequency region [36]. As presented in Figure 13, the RCT value of the S/CeO_2_/RGO cathode before cycling is 90, which is lower than that of the S/RGO cathode (120). This phenomenon indicates that the CeO_2_ particles in spherical RGO can dramatically promote charge transportation during the redox reactions. Therefore, the specific discharge capacity and the rate performance of the S/CeO_2_/RGO cathode will be considerably enhanced.

As shown in Table 1, the performance of 3D S/CeO_2_/RGO cathode is compared with other reported results. The results show that the prepared 3D S/CeO_2_/RGO cathode has good cycling performance. The discharge specific capacity is stable at 0.1 C, and the decay rate remains at 0.25% for 200 cycles.

## 4. Conclusions

The 3D S/CeO_2_/RGO composite materials were successfully synthesized via spray drying. Since this is a very simple synthesis route, high-throughput commercial manufacturing can easily be achieved. When S/CeO_2_/RGO composites are used for cathodes, they retain a capacity of 792 mAh g^−1^, even after 200 cycles of operation, under a current density of 0.1 C. Such excellent performance makes the S/CeO_2_/RGO composite a promising candidate for a low-cost, high-performance material for use in lithium–sulfur batteries.

## Figures and Tables

**Table 1:** Comparison of the electrochemical performances from previous reports and from our work.

Cathodes	Current Density (discharge)	Initial Discharge Capacity (mAh/g)	Discharge Capacity (after n th) (mAh/g)	Reference
SnO_2_@rGO/S	0.1 C	859	671 (50)	[37]
ZnO@S/CNT	0.16 A/g	988	942 (70)	[38]
MnO_2_@HCF/S	0.5 C	890	662 (300)	[39]
MgO@S	0.2 C	940	620 (100)	[40]
Fibrous rGO/S	0.75 A/g	710	541 (100)	[41]
3D S/CeO_2_/RGO	0.1 C	1054	792 (200)	This work

## References

[1] Deng Y., Li J., Li T., Gao X., Yuan C. (2017). Life cycle assessment of lithium sulfur battery for electricvehicles. J. Power Sources.

[2] Rosenman A., Markevich E., Salitra G., Aurbach D., Garsuch A., Chesneau F.F. (2015). Review on Li-sulfur battery systems: An integral perspective. Adv. Energy Mater..

[3] Yin Y.X., Xin S., Guo Y.G., Wan L.J. (2013). Lithium-sulfur batteries: Electrochemistry, materials, andprospects. Angew. Chem. Int. Ed..

[4] Li N., Gan F., Wang P., Chen K., Chen S., He X. (2018). In situ synthesis of 3D sulfur-dopedgraphene/sulfur as a cathode material for lithium-sulfur batteries. J. Alloys Compd..

[5] Xu T., Song J., Gordin M.L., Sohn H., Yu Z., Chen S., Wang D. (2013). Mesoporous carbon-carbon nanotube-sulfur composite microspheres for high-areal-capacity lithium-sulfur battery cathodes. ACS Appl. Mater. Interface.

[6] Kolosnitsyn V.S., Karaseva E.V. (2008). Lithium-sulfur batteries: Problems and solutions. Russ. J. Electrochem..

[7] Zhang Y., Zhao Y., Bakenov Z. (2014). A novel lithium/sulfur battery based on sulfur/graphene nanosheet composite cathode and gel polymer electrolyte. Nanoscale Res. Lett..

[8] Evers S., Nazar L.F. (2013). New approaches for high energy density lithium-sulfur battery cathodes. Acc. Chem. Res..

[9] Chen S.R., Zhai Y.P., Xu G.L., Jiang Y.X., Zhao D.Y., Li J.T., Huang L., Sun S.G. (2011). Ordered mesoporous carbon/sulfur nanocomposite of high performances as cathode for lithium–sulfur battery. Electrochim. Acta.

[10] Sun Z., Wang S., Yan L., Xiao M., Han D., Meng Y. (2016). Mesoporous carbon materials prepared from litchi shell as sulfur encapsulator for lithium-sulfur battery application. J. Power Sources.

[11] Xu Z.L., Kim J.K., Kang K. (2018). Carbon nanomaterials for advanced lithium sulfur batteries. Nano Today.

[12] Liang X., Kwok C.Y., Lodi-Marzano F., Pang Q., Cuisinier M., Huang H., Hart C.J., Houtarde D., Kaup K., Sommer H. (2016). Tuning transition metal oxide-sulfur interactions for long life lithium sulfur batteries: The “goldilocks” principle. Adv. Energy Mater..

[13] Zhang Y., Zhao Y., Yermukhambetova A., Bakenov Z., Chen P. (2013). Ternary sulfur/polyacrylonitrile/Mg_0.6_Ni_0.4_O composite cathodes for high performance lithium/sulfur batteries. J. Mater. Chem. A.

[14] Yin F., Liu X., Zhang Y., Zhao Y., Menbayeva A., Bakenov Z., Wang X. (2017). Well-dispersed sulfur anchored on interconnected polypyrrole nanofiber network as high performance cathode for lithium-sulfur batteries. Solid State Sci..

[15] Jeong T.G., Lee Y.S., Cho B.W., Kim Y.T., Jung H.G., Chung K.Y. (2018). Improved performance of dual-conducting polymer-coated sulfur composite with high sulfur utilization for lithium-sulfur batteries. J. Alloys Compd..

[16] Chen D., Yang R., Chen L., Zou Y., Ren B., Li L., Li S., Yan Y., Xu Y. (2018). One-pot fabrication of nitrogen and sulfur dual-doped graphene/sulfur cathode via microwave assisted method for long cycle-life lithium-sulfur batteries. J. Alloys Compd..

[17] Hong X., Liang J., Tang X., Yang H., Li F. (2018). Hybrid graphene album with polysulfides adsorption layer for Li-S batteries. Chem. Eng. Sci..

[18] Jiang Y., Chen F., Gao Y., Wang Y., Wang S., Gao Q., Jiao Z., Zhao B., Chen Z. (2017). Inhibiting the shuttle effect of Li-S battery with a graphene oxide coating separator: Performance improvement and mechanism study. J. Power Sources.

[19] Park G.D., Lee J., Piao Y., Kang Y.C. (2018). Mesoporous graphitic carbon-TiO_2_ composite microspheres produced by a pilot-scale spray-drying process as an efficient sulfur host material for Li-S batteries. Chem. Eng. J..

[20] Cheng H., Zhang P., Zhao P., Wang M. (2018). Polar cross-linked polystyrene as polysulfides anchor enhanced cycle performance and coulombic efficiency for lithium sulfur batteries. J. Electroanal. Chem..

[21] Ling B., Chen A., Liu W., Liu K., Hu H., Zhang J. (2018). Simply and rapidly synthesized composites of MnO_2_ nanosheets anchoring on carbon nanotubes as efficient sulfur hosts for Li-S batteries. Mater. Lett..

[22] Sun F., Wang J., Long D., Qiao W., Ling L., Lv C., Cai R. (2013). A high-rate lithium–sulfur battery assisted by nitrogen-enriched mesoporous carbons decorated with ultrafine La_2_O_3_ nanoparticles. J. Mater. Chem. A.

[23] Ding B., Shen L., Xu G., Ping N., Zhang X. (2013). Encapsulating sulfur into mesoporous TiO_2_ host as a high performance cathode for lithium–sulfur battery. Electrochim. Acta.

[24] Phokha S., Hunpratub S., Usher B., Pimsawat A., Chanlek N., Maensiri S. (2018). Effects of CeO_2_ nanoparticles on electrochemical properties of carbon/CeO_2_ composites. Appl. Sur. Sci..

[25] Zhu P., Zang J., Zhu J., Lu Y., Chen C., Jiang M., Yan C., Dirican M., Selvan R.K., Kim D. (2018). Effect of reduced graphene oxide reduction degree on the performance of polysulfide rejection in lithium-sulfur batteries. Carbon.

[26] Qian X., Jin L., Zhu L., Yao S., Rao D., Shen X., Xi X., Xiao K., Qin S. (2016). CeO_2_ nanodots decorated ketjen black for high performance lithium–sulfur batteries. RSC Adv..

[27] Ma M., Wang H., Liang S., Guo S., Zhang Y., Du X. (2017). Porous carbon-wrapped cerium oxide hollow spheres synthesized via microwave hydrothermal for long-cycle and high-rate lithium-ion batteries. Electrochim. Acta.

[28] Tessonnier J.P., Rosenthal D., Hansen T.W., Hess C., Schuster M.E., Blume R., Girgsdies F., Pfänder N., Timpe O., Su D.S. (2009). Analysis of the structure and chemical properties of some commercial carbon nanostructures. Carbon.

[29] Chakrabartty S., Mondal A., Chakrabarti P., Singh S.K., Saha A.K., Singh P. (2016). Synthesis of biocompatible TiO_2_ nanodots: Glancing angle deposition technique. J. Nanosci. Nanotechnol..

[30] Senkevich J.J., Yang G.R., Tang F., Wang G.C., Lu T.M., Cale T.S., Jezewski C., Lanford W.A. (2004). Substrate-independent sulfur-activated dielectric and barrier-layer surfaces to promote the chemisorption of highly polarizable metallorganics. Appl. Phys. A.

[31] Stankovich S., Dikin D.A., Piner R.D., Kohlhaas K.A., Kleinhammes A., Jia Y., Wu Y., Nguyen S.B.T., Ruoff R.S. (2007). Synthesis of graphene-based nanosheets via chemical reduction of exfoliated graphite oxide. Carbon.

[32] Li H., Sun L., Zhang Y., Tan T., Wang G., Bakenov Z. (2017). Enhanced cycle performance of Li/S battery with the reduced graphene oxide/activated carbon functional interlayer. J. Energy Chem..

[33] Han S., Pu X., Li X., Liu M., Li M., Feng N., Dou S., Hu W. (2017). High areal capacity of Li-S batteries enabled by freestanding CNF/rGO electrode with high loading of lithium polysulfide. Electrochim. Acta.

[34] Li X., Pan L., Wang Y., Xu C. (2016). High efficiency immobilization of sulfur on Ce-doped carbon aerogel for high performance lithium-sulfur batteries. Electrochim. Acta.

[35] Qian X., Zhao D., Jin L., Yao S., Rao D., Shen X., Zhou Y., Xi X. (2016). Separator modified by spray-dried hollow spherical cerium oxide and its application in lithium sulfur batteries. RSC Adv..

[36] Han P., Manthiram A. (2017). Boron- and nitrogen-doped reduced graphene oxide coated separators for high-performance Li-S batteries. J. Power Sources.

[37] Liu Q., Jiang Q., Jiang L., Peng J., Gao Y., Duan Z., Lu X. (2018). Preparation of SnO_2_@rGO/CNTs/S composite and application for lithium-sulfur battery cathode material. Appl. Surf. Sci..

[38] Gu X., Tong C., Wen B., Liu L., Lai C., Zhang S. (2016). Ball-milling synthesis of ZnO@sulphur/carbon nanotubes and Ni(OH)_2_@sulphur/carbon nanotubes composites for high-performance lithium-sulphur batteries. Electrochim. Acta.

[39] Li Z., Zhang J., Lou X. (2015). Hollow Carbon Nanofibers Filled with MnO_2_ Nanosheets as Efficient Sulfur Hosts for Lithium–Sulfur Batteries. Angew. Chem..

[40] Ponraj R., Kannan A., Ahn J., Kim D. (2016). Improvement of cycling performance of lithium-sulfur batteries by using magnesium oxide as a functional additive for trapping lithium polysulfide. ACS Appl. Mater. Interfaces.

[41] Zhou G., Yin L., Wang D., Li L., Pei S., Gentle I., Li F., Cheng H. (2013). Fibrous Hybrid of Graphene and Sulfur Nanocrystals for High-Performance Lithium Sulfur Batteries. ACS Nano.

